# A Rare Case of Acute Compartment Syndrome (ACS) Involving the Upper Limb in a Patient on Warfarin

**DOI:** 10.7759/cureus.31916

**Published:** 2022-11-26

**Authors:** Riddhi R Machchhar, Ankita Prasad, Kajal Ghodasara, Saniya H Patel, Pramil Cheriyath

**Affiliations:** 1 Internal Medicine, Rowan University School of Osteopathic Medicine, Stratford, USA; 2 Internal Medicine, Hackensack Meridian Ocean Medical Center, Brick, USA; 3 Internal Medicine, Ocean University Medical Center, Brick, USA; 4 Research, Hackensack Meridian Ocean Medical Center, Brick, USA; 5 Internal Medicine, Hackensack Meridian Health, Ocean Medical Center, Brick, USA

**Keywords:** compartment, warfain, venous thrombosis, fasciotomy, anterior compartment syndrome

## Abstract

Acute compartment syndrome (ACS) is an acute event characterized by increased pressure in the extremities where fascia encloses muscles, vessels, and nerves, leading to complications in tissue perfusion and, eventually, tissue necrosis and death. This is usually seen after trauma, crush injuries, and fractures. Similar events can also happen in the abdomen and lead to impaired perfusion in the abdominal organs. Hypovolemia, medications, and repeated or suboptimal diagnostic tests tend to worsen a pre-existing ACS, and the mainstay of its management is fasciotomy to prevent ischemic necrosis and rhabdomyolysis. Here we discuss a 64-year-old female with ACS involving the left upper limb, secondary to anticoagulation on warfarin and aspirin for atrial fibrillation. Her history was significant for peripheral vascular disease, above-knee amputation, and congestive heart failure. This article emphasizes the importance of early recognition and management of ACS to salvage limbs.

## Introduction

Acute compartment syndrome (ACS) is an emergent situation, and 69%-75% [1] of the cases are due to fractures and crush injuries involving limbs. Rapidly evolving tissue swelling or bleeding causes increased pressure in the enclosed muscle compartments, and intracompartmental pressures (ICPs) above 30 mmHg of diastolic pressure cause compression of nerves and blood vessels, furthering tissue necrosis and ischemia. The patient typically presents with one or a combination of the 5 "P's": pain, paresthesia, pallor, absent pulses, and poikilothermia. Management of ACS involves the removal of the occlusive dressing and supportive care. The gold-standard treatment is a fasciotomy. Failure of timely and effective treatment may lead to limb amputation [1]. Timely intervention is vital as a fasciotomy is most beneficial within 6 h of ACS onset [1-2]. This case report discusses a 64-year-old patient on warfarin and an international normalized ratio (INR) > 16, along with left forearm thrombotic veins, which complicated the timely diagnosis and management of ACS. It highlights the need for a systems-based and comprehensive approach to evolving cardiovascular and orthopedic situations.

## Case presentation

Our patient is a 64-year-old female who presented to the emergency room (ER) with abdominal pain, five episodes of black tarry stools, and bright red blood per rectum for one day. At the presentation to the ER, she was pale and lethargic; her heart rate was 106 beats per minute, her blood pressure was 82/48 mmHg, her oxygen saturation was 92% on room air, and her respiration rate was 26/min. She had two cardiac arrest episodes soon after arriving, and after resuscitation, she was intubated and moved to the critical care unit for ventilatory and pressor support.

**Figure 1:**
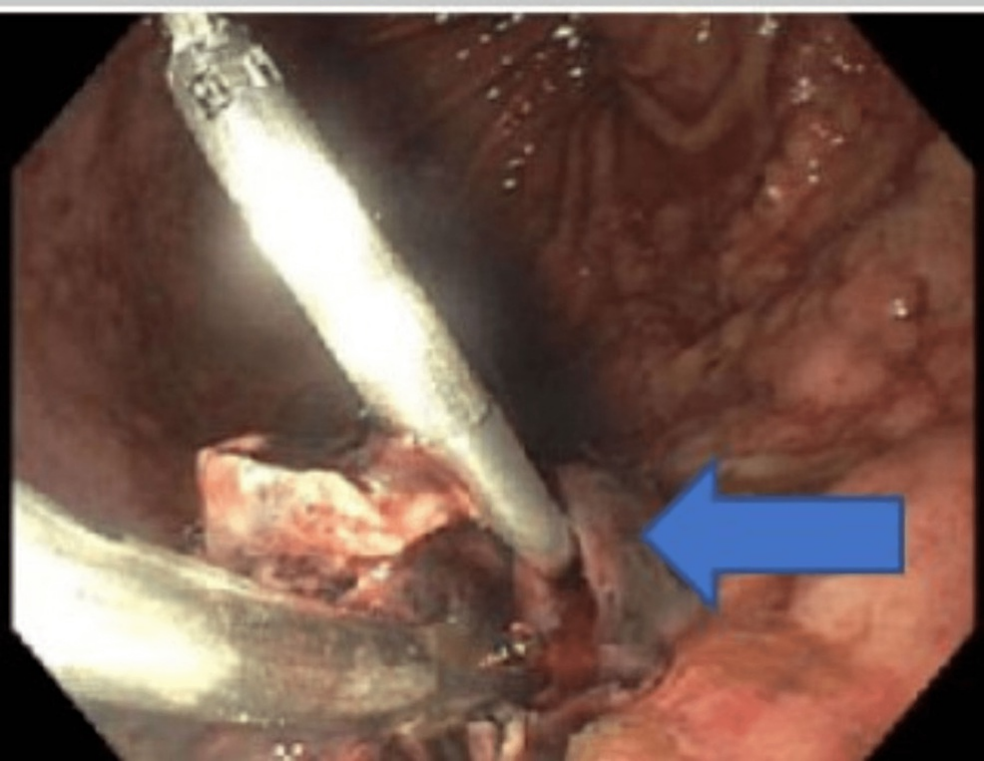
Esophoagogastroduedenoscopy images, with blue arrow pointing to clipping of the varices.

Her past medical history was significant for osteomyelitis and peripheral vascular disease status post vascular surgery and right above knee amputation done two years ago. She also had heparin-induced thrombocytopenia in the past. Her other significant medical history comprised hypertension, hypothyroidism, depression, and atrial fibrillation. She was on aspirin, warfarin (7.5 mg alternate days), metoprolol, atorvastatin, levothyroxine, escitalopram, and ferrous sulfate. She had missed her regular follow-ups after starting the warfarin for many weeks. Laboratory investigations done at presentation showed a pH of 6.85, bicarbonate of 7.6 on arterial blood gas (ABG) evaluation, hemoglobin of 5.3 g/dL (12.1-15.1 g/dL), hematocrit of 18.5 (36%-48%), and a platelet count of 350,000/mm*3 (150,000-450,000/mm*3). Her blood urea nitrogen was 54 mg/dL (6-24 mg/dL), creatinine was 2.21 mg/dL (0.6-1.1 mg/dL), serum sodium was 138 mmol/L (136-144 mmol/L), potassium was 4.9 mmol/L (3.7-5.1 mmol/L), total protein was 5.6 g/dL (6.0-8.3g/dL), albumin was 2.5 g/dL (3.4-5.4 g/dL), and a prothrombin time (PT)/INR of 187.1/16.24 on warfarin. To control the bleeding and raise hematocrit, she was given three units of packed red cells, four units of fresh frozen plasma (FFP), prothrombin complex concentrate transfusions, and a repeat PT/INR was 18.1/1.64 after these. She was also started on argatroban in appropriate doses. An esophagogastroduodenoscopy was done, and three bleeding vessels were clipped (Figure 1).

**Figure 2:**
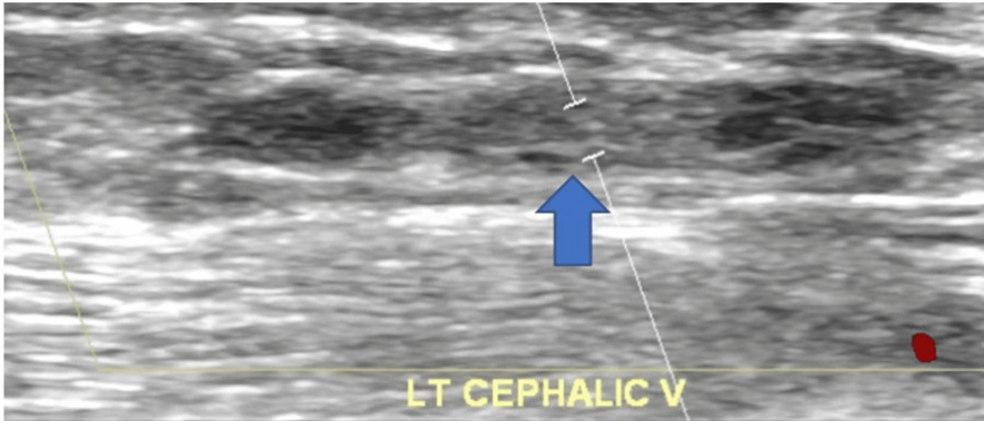
USG Doppler left cephalic vein with blue arrow showing complete thrombotic occlusion. USG, ultrasonography

**Figure 3:**
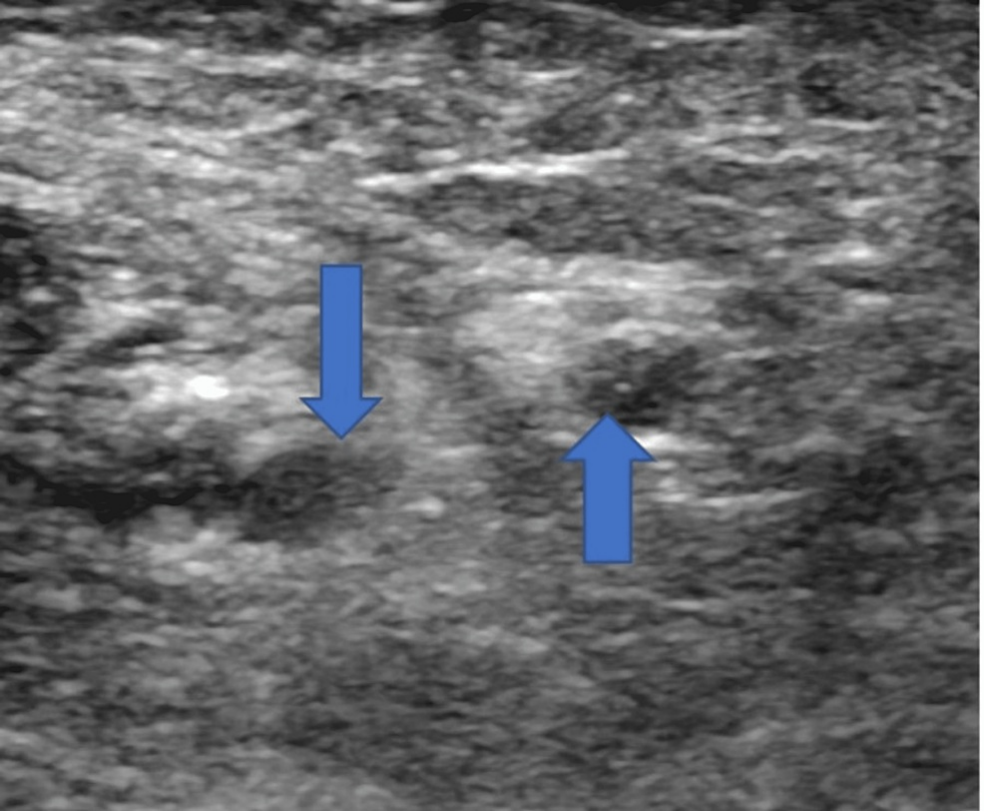
Ultrasound Doppler with blue arrow showing thrombus in left basilic vein.

Over the next two days, she developed swellings in both her arms, and her left arm and hand were cool-to-touch, mottled, and tense. A Doppler confirmed the presence of the left brachial pulse; left radial venous blood gas (VBG) revealed continued metabolic acidosis (pH=6.870), and the left antecubital (AC) IV line was removed. Within the next few hours, her left arm had cyanosis and no pulse. After 8 h, a repeat arterial and venous ultrasound (US) Doppler revealed a thrombus in her left basilic and cephalic veins (Figures 2-3). 

Brachial artery flow was present in her proximal antecubital fossa but not further downstream. The presence of a thrombus was attributed to the rapid correction of coagulopathy. She was evaluated for acute compartment syndrome (ACS) by retrieving the Stryker compartment pressure of 65 mmHg in the patient’s dorsal compartment of the left forearm. A left forearm fasciotomy and carpal tunnel release were done with the diagnosis of ACS in the left upper extremity (LUE). The fasciotomy was initially successful with the return and maintenance of the left ulnar signal. Unfortunately, flow deteriorated after one day, and the arm became unsalvageable, necessitating amputation of the left forearm and hand, which the family refused. They decided to provide comfort care in a hospice, where she passed away.

## Discussion

Acute compartment syndrome is a rapidly progressing condition seen more commonly in the lower extremities and abdomen and is less common in the upper extremities [3]. It has an overall incidence of 4.2% [4] and is reported more commonly in younger males, with 7.3 per 100,000 males and 0.7 per 100,000 females [4]. Patients developing atraumatic ACS usually have multiple comorbidities, as in our patients. They have higher chances of delayed diagnosis and treatment and, thus, complications like muscle necrosis. Compartment syndrome has been reported as a complication of anticoagulant use after minimal trauma [3]. In this case, venous punctures and venous thrombosis probably caused undiagnosed extravasation. Post-recovery ACS patients may develop residual pain, Volkmann’s contracture, mild neurological deficits, and occasionally significant cosmetic defects in the affected extremity [4]. Infection leading to sepsis and multiorgan failure is reported as the most common cause of death in patients who die from ACS [2]. In a study by Zhang et al. [5], up to 6.6% mortality was reported in ACS patients. It was more common in older people (p = 0.03), and it was associated with a higher modified Charlson Comorbidity Index (p = 0.009), higher serum potassium levels (p = 0.02), lower hemoglobin (p = 0.002), and higher lactate levels (p = 0.001) [5]. Up to 9.5% of hospitalized people with ACS have had a limb amputated [6]. This outcome is statistically significant in people with diabetes (p = 0.05), without compartment pressure measurement (p = 0.009), a longer partial thromboplastin time (PTT) (p = 0.03), and low serum albumin (p = 0.01) [6].

Anticoagulants are frequently used in patients with atrial fibrillation and are followed up with regular PT/INR, which is targeted between two and three. Failure to follow up the PT/INR in patients on anticoagulation can interfere with achieving hemostasis after trauma. When the extravasated blood fills a narrow compartment space, like the dorsal compartment of the upper limbs, the pressure can rapidly increase, causing ACS. This patient had a supratherapeutic INR of 16.24 with gastrointestinal bleeding at presentation. This complication was further compounded by undiagnosed bleeding, probably following venous cannulation and arterial puncture and a thrombus formation in the LUE, most probably due to rapid correction of the coagulopathy. ACS with extensive venous thrombosis and hematoma formation are seen occasionally. A combination of venous thrombosis, supratherapeutic anticoagulation, and venous punctures was the likely reason for raised ICPs in our patient. Even after successfully diagnosing ACS on repeated evaluations, the therapeutic window for fasciotomy was lost probably due to delayed diagnosis, and the extensive soft tissue necrosis necessitated amputation as a life-saving measure. Critically sick individuals constitute a vulnerable population whose inability to exhibit the disease's characteristic symptoms, and signs put them at risk for a compartment syndrome diagnosis that is made too late and its potentially fatal consequences. In a similar case reported by Lim and Biagio [7], a young male on warfarin developed spontaneous ACS and LUE venous thrombosis. He had a positive outcome following a successful fasciotomy. In other documented cases of ACS in the LUE, iatrogenic causes from thrombolysis were found in the case reported by Bisarya et al. [8], and repeatedly drawing arterial and venous blood samples were identified as the preceding events in the case documented by Garner et al. [9]. 

## Conclusions

This case report highlights the importance of comprehensively approaching a patient and stratifying a plan after assessing for the most devastating to most likely differential diagnosis. Our patient had ACS following arterial and venous punctures in LUE, multiple comorbidities, and supratherapeutic anticoagulation. This case reinforces the importance of special care of peripheral access and early recognition of discoloration and swelling of limbs in patients on anticoagulants to prevent such occurrences. The same applies to all sick patients who cannot complain about pain, such as small children. Timely recognition can provide early intervention and avoid adverse outcomes like prolonged ischemia, limb loss, and death. Critical care protocols for early recognition and emergent management should be in place for all peripheral venous access in anticoagulated patients. 
